# Forecasting Healthy Life Expectancy Among Chilean Community-Dwelling Older Adults With and Without Sarcopenia

**DOI:** 10.3389/fmed.2022.841810

**Published:** 2022-02-16

**Authors:** Ximena Moreno, Lydia Lera, Carlos Márquez, Cecilia Albala

**Affiliations:** ^1^Unidad de Nutrición Pública, Instituto de Nutrición y Tecnología de los Alimentos, Universidad de Chile, Santiago, Chile; ^2^Facultad de Psicología, Universidad San Sebastián, Santiago, Chile; ^3^Latin Division, Keiser University, Fort Lauderdale, FL, United States

**Keywords:** disability-free life expectancy, life expectancy, sarcopenia, gender, longitudinal studies

## Abstract

**Background:**

Sarcopenia is an important risk factor for disability and dependency at old age. The prevalence of sarcopenia among the Chilean older population is high.

**Objective:**

To estimate life expectancy, healthy life expectancy and unhealthy life expectancy among sarcopenic and non-sarcopenic older adults from Santiago, Chile.

**Methods:**

A sample of 1,897 community-dwelling older adults aged 60 years or more, living in Santiago, was observed between 5–15 years. Disability was defined as the unhealthy state, assessed through self-reported difficulties in activities of daily living. Sarcopenia was determined via HTSMayor software. Total and marginal life expectancies were estimated using the Interpolated Markov Chain method “IMaCh”.

**Results:**

At 60 years, estimated life expectancy for sarcopenic and non-sarcopenic older adults was similar (22.7 and 22.5 years, respectively). The proportion of years to be lived with disability was three times greater in sarcopenic adults, compared to non-sarcopenic people. This difference was observed up to 80 years. Non-sarcopenic women had a higher proportion of years to be lived with disabilities compared to non-sarcopenic men of the same age, but this proportion was higher among sarcopenic men, compared to sarcopenic women until 70 years of age.

**Discussion:**

People with sarcopenia expect to live a higher proportion of years with disabilities. Sarcopenic men until 70 years expected to live a higher proportion of years with disability, compared to sarcopenic women. Monitoring sarcopenia among older people may help to identify individuals with higher risk of disability onset. Future research should focus on disentangling the mechanisms explaining sex differences.

## Background

Since 2016, sarcopenia has been recognized as a condition with its own code (M62.84) in the International Classification of Disease, Tenth Revision, Clinical Modification (ICD-10CM) (1). Sarcopenia is defined as a progressive and generalized skeletal muscle disorder, characterized by loss of muscle mass, strength and function, associated with age and with an increased risk of falls, fractures, morbidity, disability, mortality, and poor quality of life (2, 3). There are also economic costs for healthcare systems, individuals and families, associated with sarcopenia, due to the increased risk of hospitalization and higher costs during hospital stay, compared to people of the same age without sarcopenia (4).

Worldwide prevalence of sarcopenia is high. A recent review concluded that different definitions resulted in wide variation of estimations, and according to studies that employed the definition of the European Working Group on Sarcopenia in Older People (EWGSOP) (5), considering low muscle mass and low muscle strength or low physical performance, the average prevalence was 9.9% and increased with increasing age, reaching 19.4% in the oldest old (6). In Chile, sarcopenia prevalence estimated with the EWGSOP algorithm is 19.1%, similar for men and women, and it is associated with age (7).

In Chile, the population aging is advanced, with the highest life expectancy (LE) at birth (80 years) of South America, along with French Guiana (8). It is important to determine if those years are lived in good health or if the additional years of life result in an expansion of morbidity among the older population (9). Considering that sarcopenia is a highly prevalent condition among older adults, and that its is associated with disability (10), it is necessary to determine the impact of sarcopenia on health expectancies.

Health expectancies integrate health status to the LE indicator, providing information with respect to the total number of years expected to be lived at a certain age, and the number of years expected to be lived in a certain health status, such as free of disability (9). Estimations of health expectancies in Chile are scarce. Previous Chilean studies reported that women at 60 years expected to live a higher proportion of years with disabilities, compared to men of the same age (11–13).

The aim of this study was to estimate LE, healthy life expectancy (HLE) and unhealthy life expectancy (ULE) among sarcopenic and non-sarcopenic older adults from Santiago, Chile.

## Methods

This is a dynamic cohort study. The sample included 1,897 people aged 60 years and older, living in the community in Santiago, Chile, who were originally recruited to the Alexandros (14) and HTS Mayor (15) projects, aimed to study disability associated with obesity and sarcopenia in Chilean older people. The Alexandros sample (*N* = 2,311) was recruited between 2003 and 2008, and the HTS Mayor sample (*N* = 169), in 2012. Baseline information was collected between 2003–2012. People who died in the following 6 months from baseline (1.5%), and those who had missing data in the outcome variables (3.8%) were excluded from the analyses. Sixty-eight people (2.7%) rejected to take part in this study. From a total of 2,281 people who were eligible and accepted to take part in this study, 384 (16.8%) were lost to follow-up (Supplementary Material 1). One thousand eight hundred ninety-seven participants were followed-up between 5–15 years (interquartile range 5.17 years), until 2017.

Data were collected via face-to-face interviews, carried out by trained interviewers at the Institute of Nutrition and Food Technology. The interviews included a structured questionnaire to gather sociodemographic information and self-reported health problems, including chronic diseases and functional status. The 5 item Geriatric Depression Scale (GDS-5) (16) was used to determine depressive symptoms. Cognitive status was assessed via the Mini-Mental State Examination (MMSE) (17) and the Functional Activities Questionnaire (FAQ) (18). Anthropometric measurements included weight, knee height, waist, hip, and calf circumference. Handgrip strength was measured in the dominant hand, with calibrated dynamometers, following the Southampton protocol. Mobility was assessed with questions about the ability to walk several blocks, climbing stairs, pushing or pulling heavy objects, lifting or carrying weights over ten 5 kg, and picking up a coin from a table. The details of the operationalization of sarcopenia are described below.

Functional status was determined according to the criteria proposed by Albala et al. (19) for the Chilean older population, considering limitation in at least one activity of daily living (ADL), or in two instrumental activities of daily living (IADL), or in three advanced activities of daily living (AADL), or a score of MMSE<13 and PFAQ>5.

Sarcopenia was defined as an adapted version of the diagnostic algorithm of the European Working group on Sarcopenia in Older People (EWGSOP1) in 2010 (5), considering low physical performance, low muscle strength, and/or low muscle mass. Participants with sarcopenia were identified by means of HTSMayor software (14). A prediction model for the Chilean population (18) was employed to estimate appendicular skeletal muscle mass (ASM):

ASM (kg) = 0.107(weight) + 0.251(knee-height) + 0.197(calf-circumference) + 0.047(dynamometry) - 0.034 (hip-circumference) + 3.4178 (male) - 0.020 (age) - 7.646;

Coefficient of determination = 0.89;

Standard Error of the Estimation = 1.346 kg.

Cut-off points of the skeletal muscle mass index (SMI = ASM/height^2^) for the Chilean population were obtained from this prediction model, resulting in 7.45 kg/m^2^ for men, and 5.88 kg/m^2^ for women (20). Muscle strength was assessed considering the best two scores obtained with a handgrip dynamometer, using the dominant hand. Cut-off points previously estimated for the Chilean population (21, 22) were employed. A combination of two physical performance tests was used to assess this dimension, since not all participants had the three meters (3 m) walking speed register. The tests used were 3 m walking speed and timed up and go (TUG) speed, in this order (23).

Mortality data were confirmed via death certificates obtained from the National Civil Registry until July 30, 2017. Vital status of all participants was known by the end of follow-up.

Descriptive analyses were carried out with Stata 15 (StataCorp.2015. Stata Statistical Software, Release 14. College Station, TX, StataCorp LP). To estimate life expectancies and disability-free life expectancies, multistate methods were employed. Three states were considered—healthy, disabled, and dead—with five possible transitions: healthy-healthy, healthy-disabled, disabled-healthy, healthy-dead, disabled-dead. The Interpolated Markov Chain (IMaCh) software was employed for the calculations (24, 25). Estimations were expressed as number of years and percentages, and 95% confidence intervals were calculated.

Cox proportional hazards models were used to estimate the association between sarcopenia and the incidence of functional limitation, including sociodemographic variables, body mass index, multimorbidity (defined as two or more self-reported chronic diseases, including high blood pressure, diabetes, coronary heart disease, stroke, chronic obstructive pulmonary disease, cancer, and arthritis), depressive symptoms, and falls. Self-reported depression was not included in this variable, due to the underdiagnosis of depression by health care providers in Chile (26). No violations of the proportional hazards' assumption were detected. Since falls and depression have been previously reported as risk factors of functional limitation (27–30), interaction terms between sarcopenia and each of these variables were tested.

Study protocols and consent forms were approved by the ethics committee of the Institute of Nutrition and Food Technology. All participants gave signed informed consent.

## Results

As observed in Table 1, the sample had a higher proportion of women (67.7%), and the mean age was 69.2 years (SD = 6.8). Baseline prevalence of sarcopenia was 22.4%, with no difference between men and women (*p* = 0.8). Functional limitation had a prevalence of 16.6% (men = 11.4%, women = 19.1%). Participants were followed-up for a median of 5.8 years, and a total of 6,358.6 person years. During the follow-up, 105 new cases of functional decline were observed, 127 people with baseline functional limitation had recovered and 492 deaths occurred.

**Table 1:** Baseline characteristics of the sample.

	**Total (*n =* 1,897)**	**Men (*n =* 612)**	**Women (*n =* 1,285)**	
**Variable**	**%**	**%**	**%**	***P*-value**
Age (years)^*^	69.2 ± 6.8	69.3 ± 6.4	69.2 ± 7.0	0.7651
Age groups				0.029
60–69.9	70.0	68.0	71.0	
70–79.9	20.5	23.7	18.9	
≥80	9.5	8.3	10.1	
Education> 8 years	40.4	41.1	40.0	0.5831
Living alone	10.0	8.9	10.5	0.290
Functional limitation	16.6	11.4	19.1	<0.001
Multimorbidity (≥2 CD)	47.4	42.8	49.6	<0.001
Depression (GDS-5)	30.9	26.3	33.0	0.0031
BMI^*^	28.6 ± 5.0	27.5 ± 4.4	29.1 ± 5.2	<0.001
Nutritional status				<0.001
Underweight	2.1	2.3	2.0	
Normal	21.0	26.0	18.6	
Overweight	43.5	47.1	41.8	
Obese	33.4	24.7	37.6	
Sarcopenia	22.4	22.1	22.6	0.804
Falls	32.6	27.8	34.8	0.003
ADL limitation ≥ 1	11.9	11.3	12.2	0.553
IADL limitation ≥ 1	26.8	31.2	24.8	0.003
MMSE	6.8	6.4	7.0	0.649

* *Mean and standard deviation*.

Table 2 shows that LE of sarcopenic and non-sarcopenic older adults at 60 years was 22.7 and 22.5 years, respectively. There were no differences in LE between both groups, but at every age, non-sarcopenic older adults had more healthy years and less ULE. Total LE was significantly longer for women than men at all ages, with the exception of participants with sarcopenia at 80 years. No significant difference was observed in total LE among sarcopenic and non-sarcopenic men. Among women, there were no differences in total LE between groups, but sarcopenic women expected to live more years with disabilities, compared to non-sarcopenic women, and had a shorter healthy life expectancy at age 70 and 80.

**Table 2:** Total life expectancy, healthy life expectancy and unhealthy life expectancy, among sarcopenic and non-sarcopenic Chilean older men and women.

	**Without sarcopenia (*****n =*** **1,472)**			**With sarcopenia (*****n =*** **425)**		
	**TLE**	HLE^*^	ULE^**^	**TLE**	HLE^*^	ULE^**^
Total						
60 years	22.5	20.5	2.0	22.7	16.8	5.8
95% CI	21.5–23.5	19.6–21.4	1.5–2.5	21.0–24.3	15.2–18.4	4.5–7.1
70 years	14.5	12.7	1.8	14.4	8.9	5.5
95% CI	13.6–15.4	11.8–13.6	1.3–2.3	13.2–15.6	7.7–10.1	4.4–6–6
80 years	8.3	6.7	1.6	7.8	3.3	4.5
95% CI	7.4–9.2	5.8–7.6	0.9–2.3	6.9–8.7	2.4–4.2	3.6–5.4
Men						
60 years	20.1	19.0	1.2	18.3	10.7	7.6
95% CI	18.7–21.6	17.5–20.3	0.7–1.8	15.4–21.2	6.7–14.8	4.0–11.2
70 years	12.1	11.1	1.0	11.4	6.4	5.0
95% CI	10.9–13.4	9.9–12.4	0.5–1.5	9.6–13.2	4.9–8.0	3.5–6.5
80 years	6.6	5.9	0.7	6.4	3.5	2.9
95% CI	5.3–7.8	4.6–7.3	0.1–1.2	5.0–7.8	2.5–4.4	2.1–3.8
Women						
60 years	23.8	21.5	2.4	25.6	19.5	6.1
95% CI	22.5–25.2	20.2–22.8	1.6–3.1	23.8–26.6	17.7–21.4	4.7–7.6
70 years	15.7	13.5	2.2	16.4	10.6	5.8
95% CI	14.5–17.0	12.3–14.7	1.4–3.0	14.9–17.9	9.0–12.2	4.5–7.1
80 years	9.2	7.2	2.0	8.8	4.1	4.8
95% CI	7.9–10.4	5.9–8.4	1.1–2.9	7.6–10.1	2.8–5.3	3.6–5.9

* *Healthy life expectancy (without disability)*.

** *Unhealthy life expectancy (with disability)*.

**Figure 1:**
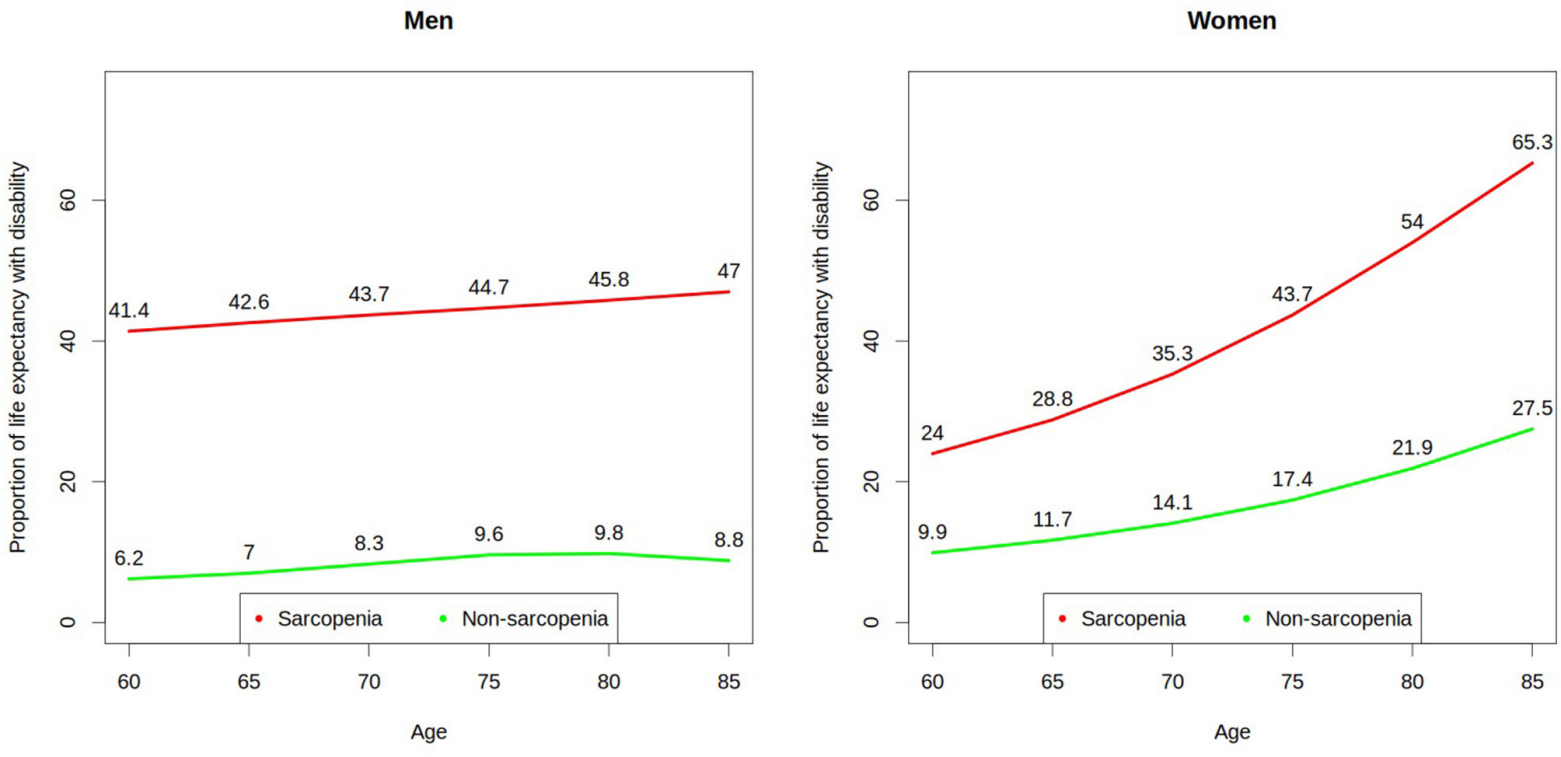
Proportion of life expectancy to be lived with disability by Chilean older men and women with and without baseline sarcopenia.

As observed in Figure 1, sarcopenic men had a higher proportion of expected ULE, compared to non-sarcopenic men, with a proportion of ULE 6.6 times higher at 60 years, 5.3 times higher at 70 and 4.7 times higher at 80 years. The proportion of ULE among sarcopenic women was 2.4 times higher at age 60, 2.5 times higher at age 70 and 4.0 times higher at age 80, compared to non-sarcopenic women. Sarcopenic men at 60 and 70 years of age had a higher proportion of expected ULE, compared to sarcopenic women of the same age, with a difference of 17.4 and 8.4 percent points, at the respective age. On the contrary, among non-sarcopenic people at ages 69, 70, and 80, a higher proportion of LE was expected to be lived with disabilities among women, compared to men (3.7, 5.8, and 12.1 percent point, respectively).

Cox proportional hazards models showed that sarcopenia and age were the only variables associated with the incidence of functional limitation (Table 3). Interaction terms of sarcopenia with falls and with depression were not significant.

**Table 3:** Hazard ratio of functional limitation among Chilean older people from the Alexandros and HTS Mayor studies, 2003–2017.

	**Model 1**	**Model 2**	**Model 3**	**Model 4**
	**HR (95% CI)**	**HR (95% CI)**	**HR (95% CI)**	**HR (95% CI)**
Sarcopenia	3.40 (2.11–5.49)	3.65 (2.02–6.62)	3.69 (1.99–6.86)	3.85 (2.05–7.22)
Age (years)	1.07 (1.03–1.11)	1.07 (1.03–1.11)	1.07 (1.03–1.11)	1.07 (1.03–1.11)
Women	0.80 (0.49–1.31)	0.79 (0.49–1.30)	0.88 (0.52–1.48)	0.82 (0.48–1.40)
Nutritional state				
BMI <20		1.06 (0.39–2.86)	0.80 (0.25–2.52)	0.78 (0.25–2.47)
BMI: 25–29.9		0.94 (0.54–1.64)	0.97 (0.54–1.74)	0.97 (0.54–1.74)
BMI: ≥30		1.17 (0.54–2.53)	1.22 (0.55–2.70)	1.22 (0.55–2.72)
Multimorbidity^*^			0.99 (0.61–1.61)	0.98 (0.60–1.60)
Depression^**^			1.10 (0.66–1.85)	1.04 (0.61–1.75)
Falls				1.43 (0.87–2.37)

* *≥2 chronic diseases*;

** *GDS-5*.

## Discussion

Our study found no difference in LE between sarcopenic and non-sarcopenic older people, but more ULE among the former. In absolute terms, at 60 years of age, sarcopenic men expected to live more years with disability, compared to non-sarcopenic men and women with or without sarcopenia. From 70 years, sarcopenic women expected to live more years with disability, compared to the other groups. With respect to the proportion of ULE, sarcopenic men expected to live a higher proportion of LE with disabilities, up to the age of 70 years.

According to our results, baseline sarcopenia had a differential impact on HLE and ULE of men and women. Sarcopenic men had a shorter HLE and more ULE, compared to sarcopenic women. This suggests that sarcopenia could be associated with a higher and earlier onset of disability among older men. A Brazilian study found that sarcopenia was associated with osteoporosis among older men, but not women (31). Among a Japanese sample without baseline restrictions in ADL, almost twice as much sarcopenic men (38.8%) as sarcopenic women (18.8%) developed restrictions in ADL (32).

A previous study found that Chilean older women had a higher proportion of ULE, compared to men of the same age (10). However, the results of the present study show the importance of considering baseline sarcopenia to describe disability trajectories of older men and women. Women with sarcopenia had a higher proportion of ULE, compared to non-sarcopenic women. Sarcopenic older men, for their part, expected to live a greater proportion of their total LE with disabilities, compared not only to non-sarcopenic men, but to sarcopenic women as well.

Genetic, nutritional, physical activity and age-related factors are associated with the onset of sarcopenia (33). Two systematic reviews have concluded that physical activity is a protective factor against sarcopenia in men and women (34, 35). Nevertheless, some studies suggest that the effect of physical activity on sarcopenia onset and progression differs between sexes (36, 37). Rivera et al. (38) found that age and physical activity were related with muscle volume and performance in men, but not in women. According to those results, sedentarism during the life-course resulted in loss of muscular mass among men, with a negative impact on function. In the case of older women, it was observed that muscular performance and functionality was preserved despite the sarcopenic process. The role of physical activity on different trajectories of disability among sarcopenic older men and women should be further studied. Also, other potential factors associated with these differences, which act as protective factors for women or negatively affect men, should be elucidated.

This study has some limitations that should be considered. ASM was estimated by an anthropometric equation instead of dual-energy X-ray absorptiometry (DXA), which is considered the gold standard to measure body composition (39). However, this equation and DXA had a high concordance correlation coefficient (0.94) in a previous Chilean study (40). On the other hand, considering the technical difficulties to assess muscle mass and quality, and the ability of muscle strength and physical performance to predict adverse outcomes, these latter measures of physical performance are primarily used (2, 4). The sample size affected the precision of our estimations, particularly in stratified analyses and for older ages. Nevertheless, we found significant differences between groups. The participants of the study were recruited among older people living in Santiago. Hence, our results are not representative at a national level. Also, the sample did not include people living in rural areas. According to previous research, Chilean older people living in rural areas have worse health status and higher levels of disability (41). Lastly, attrition bias cannot be ruled out, which could have affected out estimations, in case the distribution of sarcopenia or disability incidence varied between the participants in our study and those lost to follow-up. A higher or lower incidence of disability among men or women with or without sarcopenia who were not followed-up, could result in over or underestimation of years to be lived with disability, in one or several of the groups. This limitation should be taken into account when interpreting the results.

In conclusion, our results stress the importance of monitoring sarcopenia among older adults, to identify those individuals at a greater risk of disability onset. Sex differences observed in disability trajectories among sarcopenic older people should be disentangled by future research.

## Data Availability Statement

The raw data supporting the conclusions of this article will be made available by the authors, without undue reservation.

## Ethics Statement

The studies involving human participants were reviewed and approved by Ethics Committee of the Institute of Nutrition and Food Technology. The patients/participants provided their written informed consent to participate in this study.

## Author Contributions

LL and CA designed the primary studies. CM coordinated the data collection. LL and XM designed the secondary analyses that are reported here and wrote the draft of the manuscript. LL performed the statistical analyses. All authors revised, commented, and approved the final version of the manuscript.

## Funding

This research was supported by the Chilean National Fund for Scientific and Technological Development (Fondecyt grant 1130947 and Fondef grant 15I10053). The funders had no role in study design, data collection and analysis, decision to publish, or preparation of the manuscript.

## Conflict of Interest

The authors declare that the research was conducted in the absence of any commercial or financial relationships that could be construed as a potential conflict of interest.

## Publisher's Note

All claims expressed in this article are solely those of the authors and do not necessarily represent those of their affiliated organizations, or those of the publisher, the editors and the reviewers. Any product that may be evaluated in this article, or claim that may be made by its manufacturer, is not guaranteed or endorsed by the publisher.
