# Supplementary figures and images for: Forecasting Healthy Life Expectancy Among Chilean Community-Dwelling Older Adults With and Without Sarcopenia
Source: Front Med (Lausanne). 2022 Feb 16;9:841810. doi: 10.3389/fmed.2022.841810 (PMC8889119; doi:10.3389/fmed.2022.841810)

Flow chart of the sample inclusion, exclusion, and loss to follow-up

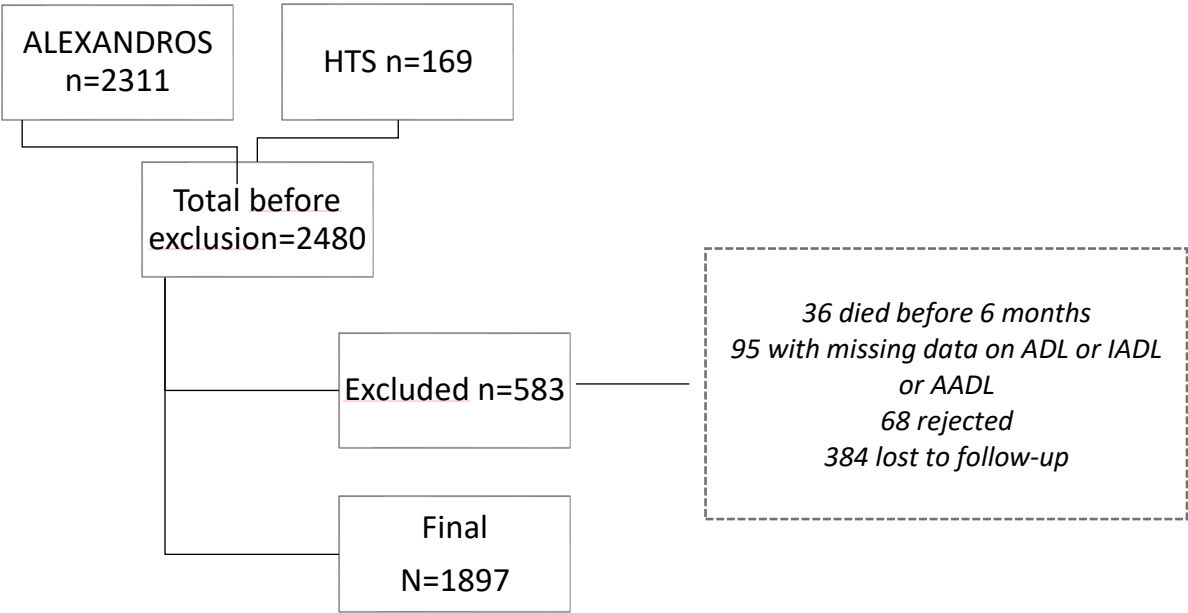

Supplement: Supplementary file 1 [file Image_1.pdf]
